# FNDC5/Irisin Is Not Only a Myokine but Also an Adipokine

**DOI:** 10.1371/journal.pone.0060563

**Published:** 2013-04-11

**Authors:** Arturo Roca-Rivada, Cecilia Castelao, Lucía L. Senin, María O. Landrove, Javier Baltar, Ana Belén Crujeiras, Luisa María Seoane, Felipe F. Casanueva, María Pardo

**Affiliations:** 1 Grupo Obesidómica, Laboratorio de Endocrinología Molecular y Celular, Instituto de Investigación Sanitaria de Santiago de Compostela (IDIS), Complexo Hospitalario Universitario de Santiago (CHUS/SERGAS), Santiago de Compostela, Spain; 2 Grupo Fisiopatología Endocrina, Laboratorio de Endocrinología Molecular y Celular, Instituto de Investigación Sanitaria de Santiago de Compostela, Complexo Hospitalario Universitario de Santiago (CHUS/SERGAS), Santiago de Compostela, Spain; 3 CIBER Fisiopatología Obesidad y Nutrición (CB06/03), Instituto de Salud Carlos III, Santiago de Compostela, Spain; 4 Servicio de Cirugía General, Complexo Hospitalario Universitario de Santiago (CHUS/SERGAS), Santiago de Compostela, Spain; 5 Laboratorio de Endocrinología Molecular y Celular, Instituto de Investigación Sanitaria de Santiago (IDIS), Complejo Hospitalario de Santiago (CHUS/SERGAS), Santiago de Compostela, Spain; University of Cordoba, Spain

## Abstract

Exercise provides clear beneficial effects for the prevention of numerous diseases. However, many of the molecular events responsible for the curative and protective role of exercise remain elusive. The recent discovery of FNDC5/irisin protein that is liberated by muscle tissue in response to exercise might be an important finding with regard to this unsolved mechanism. The most striking aspect of this myokine is its alleged capacity to drive brown-fat development of white fat and thermogenesis. However, the nature and secretion form of this new protein is controversial. The present study reveals that rat skeletal muscle secretes a 25 kDa form of FNDC5, while the 12 kDa/irisin theoretical peptide was not detected. More importantly, this study is the first to reveal that white adipose tissue (WAT) also secretes FNDC5; hence, it may also behave as an adipokine. Our data using rat adipose tissue explants secretomes proves that visceral adipose tissue (VAT), and especially subcutaneous adipose tissue (SAT), express and secrete FNDC5. We also show that short-term periods of endurance exercise training induced FNDC5 secretion by SAT and VAT. Moreover, we observed that WAT significantly reduced FNDC5 secretion in fasting animals. Interestingly, WAT of obese animals over-secreted this hormone, which might suggest a type of resistance. Because 72% of circulating FNDC5/irisin was previously attributed to muscle secretion, our findings suggest a muscle-adipose tissue crosstalk through a regulatory feedback mechanism.

## Introduction

Last January 2012 Boström and colleagues identified a new muscle-tissue-secreted peptide termed Irisin [1]. Interestingly, this myokine, whose secretion depends on the PGC1α transcriptional co-activator, seems to be capable to induce white adipose tissue (WAT) browning. Because the function of brown adipose tissue (BAT) is specialized to dissipate chemical energy as heat, the relevance of this finding if further confirmed, would be promising. Recent reports using 18 fluoro-labeled 2-deoxy-glucose positron emission tomography (18FDG-PET) have shown that, contrary to previously thought, normal adult humans have distinct brown fat deposits. Moreover, the thermogenic activity of this tissue is inversely correlated with overall adiposity, which suggests that variations in the amount or activity of BAT are related to the susceptibility for weight gain in humans [2]–[6]. Thus, BAT has recently acquired increasing relevance due to its potential role in the defense against obesity and obesity-associated disorders [7]. However, a current controversy has arisen concerning FNDC5/irisin and its expression in humans who exercise as well as its true relationship with metabolic status [8].

The transcriptional co-activator PGC1α is involved in many biological programs related to energy metabolism. PGC1α was initially implicated in the expression of UCP-1 and thermogenesis in brown fat through PPAR-γ [9] as well as the control of mitochondrial biogenesis and oxidative metabolism in many cell types. More recently, it was shown that PGC1α is induced in muscle by exercise stimulating several of the beneficial effects of exercise in this tissue [10]. Consequently, mice with transgenically increased PGC1α in their muscles are resistant to age-related obesity and diabetes [11]. Built on these premises, Boström's work reveals that exercise and PGC1α stimulates the expression of the FNDC5 gene in muscle. This gene encodes a Type I membrane protein that might be proteolytically processed to form the secreted myokine irisin. However, these authors were unable to detect the 12 kDa soluble form of irisin in their experiments. Relevantly, they showed that treating primary subcutaneous white adipocytes during differentiation with Fndc5 induced the activation of browning and thermogenic genes such as UCP1 in vitro. Furthermore, they showed that FNDC5/irisin was significantly elevated in plasma after endurance exercise in mice and humans and that circulating increments of this myokine forced with adenoviral vectors that expressed the full length of FNDC5 increased energy expenditure in mice with no changes in movement or food intake [1].

Given the relevance of the FNDC5/irisin discovery as well as its controversy and potential functions, a need to obtain additional information regarding the nature of this hormone and its precise role in energy homeostasis, including its participation in food-related disorders, exists. We believe that the present study sheds light about FNDC5 secretion from skeletal muscle and more relevantly, we set a new paradigm by demonstrating that FNDC5/irisin is not just a myokine liberated by muscle tissue but also an adipokine released by white and to a lesser extent, by brown adipose tissue. Interestingly, we show that adipose-FNDC5/irisin expression and secretion varies according to the type of fat depot and that exercise and nutritional status influence its secretion. Moreover, we describe its deregulation in food-related disorders.

## Materials and Methods

### Ethics Statement

The authors of this manuscript declare that the animal work in this study was performed according to the protocols approved by the Animal Care Committee of Santiago de Compostela University (Santiago de Compostela, Spain). Furthermore, the human adipose tissue specimen was obtained with written informed consent approved for this particular study by the Comité Ético de Investigación Clínica de Galicia – CEIC de Galicia (Spain) according to the Declaration of Helsinki.

### Animal Models

Male Sprague Dawley rats (160 g) were housed in 12-h light/12-h dark cycles with free access to a standard chow diet and water. After 5 days of acclimatization, weight-matched animals were assigned to one the following experimental groups (n = 5 per group): a) control ad libitum, b) 36 hours fasting, c) re-feed (36 hours fasting followed by 15 minutes of feeding), d) exercise (ad libitum with free access to the activity wheel for 1 or 3 weeks), e) control activity-based anorexia (ABA) and f) ABA as previously described and listed in Table S1 [12], [13]. The body weight, food intake, daily wheel turns, body composition and hormonal determination (ghrelin, leptin and HOMA) of these animals were previously characterized [13]. Diet-induced obesity (DIO) animals (200 g) were fed with a 60% high fat diet D12492 (Research Diets, NJ) over 9 weeks, and age-matched lean rats were used as a control. Male obese Zucker rats (fa/fa, n = 10) and their control counterparts, lean Zucker rats (fa/-, n = 10), were purchased from Charles River laboratories (Barcelona, Spain) at 10 weeks of age and were fed chow ad libitum for 12 weeks. Animals were euthanized by decapitation; their blood was collected, and their tissues were surgically excised.

### Rat muscle/adipose tissues and secretomes processing

The soleus and gastrocnemius muscles, the subcutaneous adipose tissue from the skin of the hips, the visceral fat located inside the peritoneal cavity around the internal organs and the brown (interscapular area) fat locations were processed for secretome collection based on a previously optimized protocol to minimize tissue damage [14], [15]. Briefly, tissues (n = 5 from each muscle type/fat depot) were carefully excised minimizing rat hair contamination as much as possible and transported from the animal house operating room to the laboratory in a sterile Krebs – Ringer-Hepes (KRH) buffer with penicillin (100 U/ml) and streptomycin (100 μg/ml) at room temperature. The tissues were processed to eliminate any contaminants and washed thoroughly in KRH under sterile conditions in a flow laminar hood. Adipose tissue pieces were centrifuged in a 25 ml tube with 20 ml of KRH for 5 minutes at room temperature to remove blood cells and cell debris. Fat pieces of approximately 2 g were incubated in 6 well cell culture dishes (Iwaki, Tokio, Japan) in 4 ml of serum-free medium that was changed twice every two hours and again after 16 hours. Finally, fresh 4 ml of serum/phenol red free DMEM medium was added and incubated for 24 hours. Whole muscle tissues were incubated in 6 well cell culture dishes (Iwaki, Tokyo, Japan) with 5 ml of serum-free medium that was changed twice every two hours and again after 16 hours. Finally, 3 ml of fresh serum/phenol red free DMEM medium was added and incubated for 12 hours. Secretomes were immediately processed for sample concentration (corrected per gram of tissue) by ultra centrifugation units (Amicon Ultra 3-kDa cut off, Millipore, Billerica, USA). A fragment of each adipose depot type and muscles from opposite legs were frozen at −80°C for western blot and quantitative real-time PCR (QRT-PCR) studies.

### Immunoblotting

Protein extracts from whole tissue samples and secretomes were processed as previously described [13], [14]. Briefly, proteins of whole tissue samples were extracted by homogenization using a TissueLyser II (QIAGEN, Tokio, Japan) in cold RIPA buffer [200 mM Tris/HCl (pH 7.4), 130 mM NaCl, 10% (v/v) glycerol, 0.1% (v/v) SDS, 1% (v/v) Triton X-100, 10 mM MgCl2] with anti-proteases and anti-phosphatases (Sigma-Aldrich; St Louis, MO). Homogenates were centrifuged for 30 minutes at 18,000 g in a microfuge at 4°C, and supernatants were frozen at −80°C. Twenty-five μg of adipose tissue secreted proteins, 25 μg of whole tissue and 5 µl of blood plasma from three independent experiments were separated in 12% SDS-PAGE gels and electroblotted onto nitrocellulose membranes as previously described [13]. Equal loading was confirmed by membrane staining with Ponceau S (Sigma-Aldrich; St Louis, MO) in the case of secretome protein extracts, or by measuring the amount of GAPDH in whole tissue protein extracts. Primary anti-FNDC5, anti-UCP-1 and anti-PGC1 were purchased from Abcam (Cambridge, UK); anti-Irisin was purchased from Phoenix Pharmaceuticals Inc. (CA, USA); anti-IL6 and anti-Adiponectin were purchased from Santa Cruz Biotechnology Inc. (CA, USA); anti-GAPDH was purchased from Life Technologies Ltd (Paisley, UK). Data were expressed as percentages corrected towards GADPH (arbitrary units) in Western blots with mean ± SEM. Data analyses were conducted using GraphPad Prism 5 software with a Mann-Whitney U test in which * p<0.05 and ** p<0.01 were considered significant and very significant, respectively.

### Human adipose tissue acquisition

Human adipose tissue was obtained from an obese patient (body mass index >35) who underwent laparoscopic gastrectomy surgery. The visceral fat was located in the hypogastric region around the internal organs, and the subcutaneous fat was located in the mesogastric region. The tissues were transported from the operating room to the laboratory in sterile KRH buffer with penicillin (100 U/ml) and streptomycin (100 μg/ml) and processed as described above for the rat adipose tissue.

### Isolation of mature adipocytes and stromo-vascular fraction (SVF)

Five g of rat adipose tissue from the visceral and subcutaneous locations was washed three to four times with KRH buffer, cut in pieces less than 1 mg and resuspended in an equal volume of 0.2% pre-warmed (37°C) collagenase Type I (Gibco, CA). This tissue was placed in an orbital mixer at 37°C with continuous agitation for 90 minutes. Then, it was centrifuged for 5 minutes at 400 g at room temperature. The supernatant that contained mature adipocytes was recollected. The pellet was identified as the SVF.

### Cell culture

The cell line 3T3-L1 was grown in DMEM with 10% fetal bovine serum (FBS), 100 U/ml of penicillin and 100 μg/ml of streptomycin until differentiation to adipocytes as previously described [16].

### RNA isolation and quantitative real-time PCR

Total RNA was isolated from adipose tissue from five independent experiments (i.e., different animals) using Trizol (Invitrogen, CA, USA) according to the manufactureŕs recommendations. Quantitative real-time PCR was performed using a Real Time PCR Systems Step One Plus (Applied Biosystems) with specific Taqman qRT-PCR primers as described elsewhere [17]. The levels of FNDC5 and PGC1 were normalized for the gene-expression analysis using hypoxanthine phosphoribosyltransferase 1 (HPRT1) rRNA (TaqMan: Applied Biosystems) as a housekeeping gene and were expressed with respect to the average value in the control group.

## Results

### White adipose tissue secretes FNDC5/irisin, which is modulated by exercise and nutritional status

**Figure 1:**
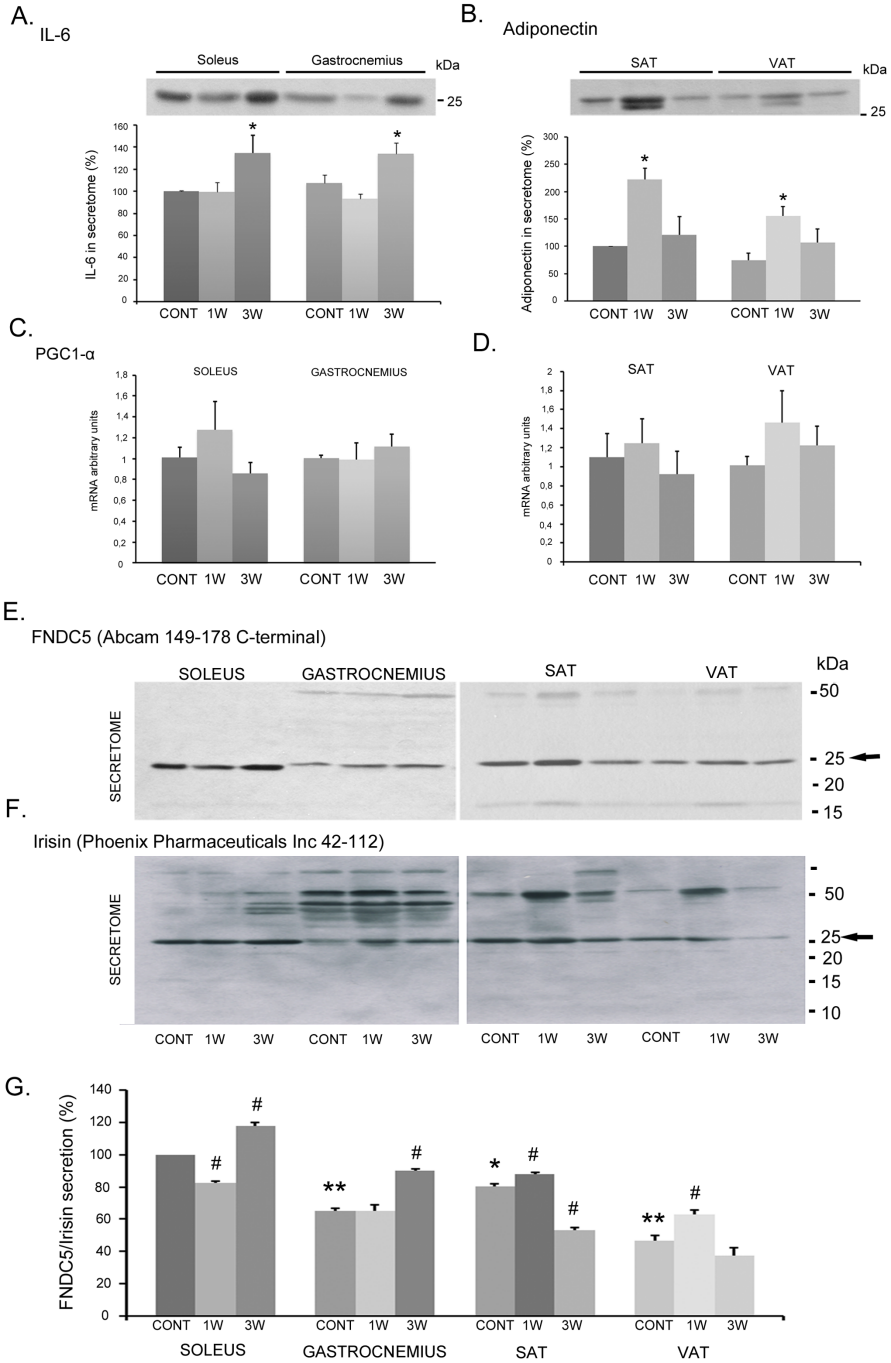
Muscle and adipose tissue sample viability and FNDC5 secretion characterization. The presence of IL-6 and adiponectin in muscle (oxidative-soleus and glycolytic-gastrocnemius) and adipose tissue (subcutaneous and visceral) secretomes assessed by western blot are shown in corresponding histograms for band quantification (A and B). PGC1α expression was tested in the same samples by real-time PCR (C and D). The presence of whole FNDC5 (E) and the soluble form of irisin (F) in muscle and adipose tissues are shown in representative images. Histograms of the band quantification from at least 3 independent experiments are shown, and significance is indicated with regard to the control soleus (*p<0.05; **p<0.01) or towards its own tissue control (^#^p<0.05) (G). CONT: control ad libitum animals; 1 and 3 W: 1 and 3 weeks of exercise training.

While characterizing FNDC5/irisin myokine regulation under different nutritional and pathological situations, we discovered that rat adipose tissue is able to express and secrete this novel peptide. Whole tissue and secretome samples from rat skeletal muscle and white adipose tissues were collected as indicated in the methods section following protocols previously described by our group [14], [18] and assessed for FNDC5/irisin secretion and expression. To confirm the viability and nature of the collected secretomes, we first tested the secretion of IL-6 and adiponectin as reference myokine and adipokine, respectively (Figures 1A, B). As expected, both gastrocnemius and soleus tissues showed IL-6 secretion that significantly increased with exercise [19] (Figure 1A); on the other hand, we confirmed that subcutaneous adipose tissue secretes more adiponectin than visceral fat [20] and that this secretion significantly increased after one week of exercise [21] (Figure 1B). Next, we examined PGC1α mRNA expression and observed that no significant differences existed among samples in either muscle or adipose tissues (Figure 1C, D). Nevertheless, one week of exercise moderately increased PGC1α in subcutaneous and visceral adipose tissues.

Once the secretomes were characterized, we analyzed FNDC5/irisin secretion levels by immunodetection among the same secretome samples (Figure 1E–F). Because the molecular weight of the secreted form of FNDC5/irisin remains controversial and not well characterized, we analyzed muscle and adipose tissue secretomes using an anti-FNDC5 antibody directed against the theoretically non-secreted portion of the protein (amino acids 149–178 from Abcam) and an anti-Irisin antibody directed against the predicted 12 kDa excised form (amino acids 42–112 from Phoenix Pharmaceuticals). Figures 1E and F show the full immunoblot picture where a predominant band of 25 kDa was detected in muscle and adipose tissue secretomes with both antibodies. Surprisingly, we were unable to detect a strong 12 kDa band signal with the anti-irisin antibody (Figure 1F). However, the anti-irisin antibody detected a strong band for the 25 kDa complete protein with an expression pattern identical to the immunoblot performed with the anti-FNDC5 antibody (indicated with arrows in Figure 1E and F). In addition, both antibodies were able to detect extra bands with higher and, to a lesser extent, lower molecular weights. The densitometry of 5 independent experiments expressed as percentages in reference to the soleus 25 kDa FNDC5/irisin secretion levels is shown in Figure 1G. Interestingly, we observed that, under basal conditions, slow-oxidative fiber-type muscle (soleus) secretes approximately 40% more FNDC5/irisin than fast-glycolytic fiber-type muscle (gastrocnemius), 20% more than subcutaneous adipose tissue, and approximately 60% more than visceral adipose tissue (Figure 1G). Confirming the observations in humans [1], both the soleus and gastrocnemius muscles showed a significant increase of FNDC5/Irisin secretion after three weeks of voluntary exercise. Excitingly, subcutaneous and visceral adipose tissues showed increased secretion after only one week of exercise (Figure 1G).

**Figure 2:**
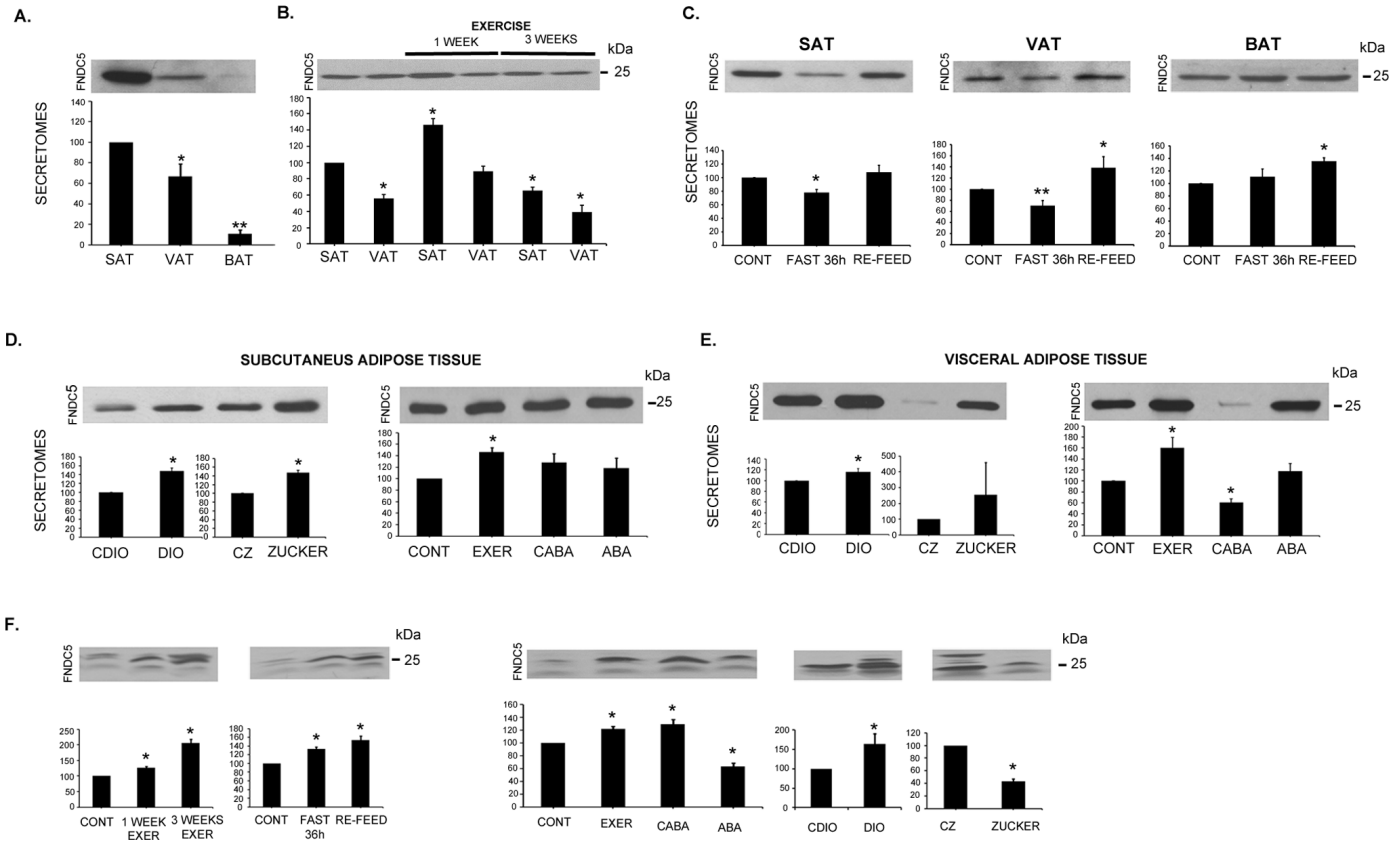
Adipose tissue secretion of FNDC5/irisin. FNDC5/irisin secretion was assessed in adipose tissue secretomes from SAT, VAT and BAT under ad libitum conditions (A); in SAT and VAT secretomes from animals after one and three weeks of exercise training (B); and in SAT, VAT and BAT from animals with 36-h food restriction or re-feed after 15 minutes (C). FNDC5/irisin SAT and VAT secretion from the DIO and Zucker animal models and anorexic animals are also shown (D, E). Irisin circulating levels for all previous situations are shown taking the most intense band at 25 kDa (F). Representative images of western blots and histograms of band quantifications from at least 3 independent experiments are shown. The results are represented as percentages with regard to each control as well as significance. In the situation of 3 weeks of exercise, results are compared with 1 week of exercise (*p<0.05; **p<0.01). SAT: subcutaneous adipose tissue; VAT: visceral adipose tissue; BAT: brown adipose tissue; DIO: diet induced obesity; CDIO: control DIO; CZ (fa/-): control Zucker; Zucker: fa/fa; CONT: control; EXER: exercise; CABA: control ABA; ABA: activity based anorexia.

**Figure 3:**
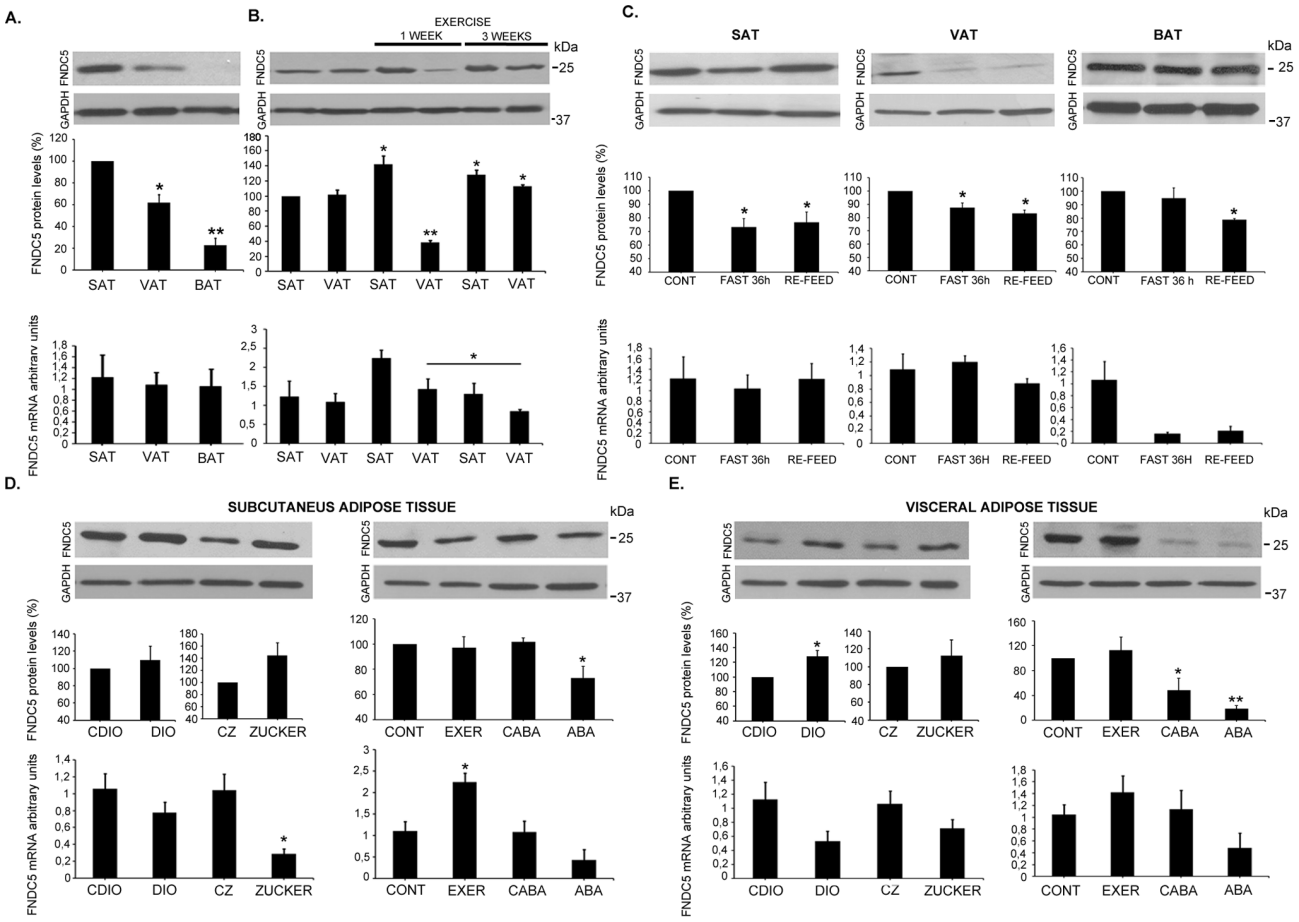
Adipose tissue expression of FNDC5. FNDC5 expression was assessed in whole adipose tissue using QRT-PCR and western blot in the same secretion study samples shown in Figure 2. Representative images of western blots and histograms of band quantification towards GAPDH from at least 3 independent experiments are shown. Significant protein results are represented as percentages with regard to each control as well as statistically significance (*p<0.05; **p<0.01). QRT-PCR results are shown as arbitrary units and compared with each control (*p<0.05). SAT: subcutaneous adipose tissue; VAT: visceral adipose tissue; BAT: brown adipose tissue; DIO: diet induced obesity; CDIO: control DIO; CZ (fa/-): control Zucker; Zucker: fa/fa; CONT: control; EXER: exercise; CABA: control ABA; ABA: activity based anorexia.

Given the interesting finding linking adipose tissue with FNDC5/irisin, we decided to better characterize the expression and secretion of this protein in adipose tissue under different nutritional and pathological situations. When comparing adipose tissues from different anatomical localizations, we observed that SAT expressed and secreted 40% more FNDC5/irisin than VAT; nevertheless, BAT barely expressed and secreted FNDC5/irisin compared with SAT and VAT under the ad libitum condition (Figures 2A and 3A). In addition, we confirmed that one week of endurance exercise significantly increased FNDC5/irisin expression and secretion in SAT (46%) and VAT (36%); however, after three weeks of training, FNDC5/irisin secretion significantly diminished in both fat deposits compared with non-exercised controls (Figure 2B). Moreover, we observed that 36 hours of fasting significantly decreased FNDC5/irisin liberation and expression in both SAT and VAT (Figure 2C and 3C); this effect reverted in both tissues secretion after re-feeding fasting animals for 15 minutes (Figure 2C). Conversely, fasting did not affect FNDC5/irisin secretion in BAT (Figure 2C).

### Adipose FNDC5/irisin secretion under pathological situations

Because knowing whether adipose FNDC5/irisin plays a role in pathological situations is of interest, we examined FNDC5/irisin secretion and expression in obese animals. Rats with either diet-induced obesity (DIO) or genetic obesity (Zucker rats) showed a significant increase of FNDC5/irisin secretion in both SAT and VAT tissues compared with their lean counterparts (Figure 2D and E). Furthermore, we studied a well-characterized rat model for anorexia, activity based anorexia (ABA), which also included control animals with voluntary exercise (EXER) and food restriction (CABA) [14]. FNDC5/irisin detection in the SAT and VAT of anorexic animals did not reveal differences compared with ad libitum control animals (Figure 2D and E). Control animals that voluntarily exercised for one week showed a significant increase of FNDC5/irisin secretion in their SAT and VAT as described above (Figure 2D and E). Moreover, the VAT from CABA animals under the same fasting regime as ABA animals but without exercise, showed considerably less FNDC5/irisin liberation; this effect was reverted by exercise in VAT from ABA animals (Figure 2E). The FNDC5 protein expression and mRNA levels were assessed for all experimental settings and are shown in Figure 3.

### Circulating FNDC5/irisin

FNDC5/Irisin circulating levels were assessed in plasma samples from all the animals studied above (Figure 2F). This analysis revealed a significant increase in FNDC5/irisin levels after 1 and 3 weeks of exercise, after 36 hours of fasting and after 15 minutes of re-feeding. Corroborating this finding, we detected a significant increase of FNDC5/irisin levels in ABA control exercised animals (EXER) and CABA. However, DIO and Zucker rats had opposite circulating levels of FNDC5/irisin; whereas DIO animals showed a significant increase, Zucker rats showed significantly diminished levels.

### Adipose tissue “browning”

**Figure 4:**
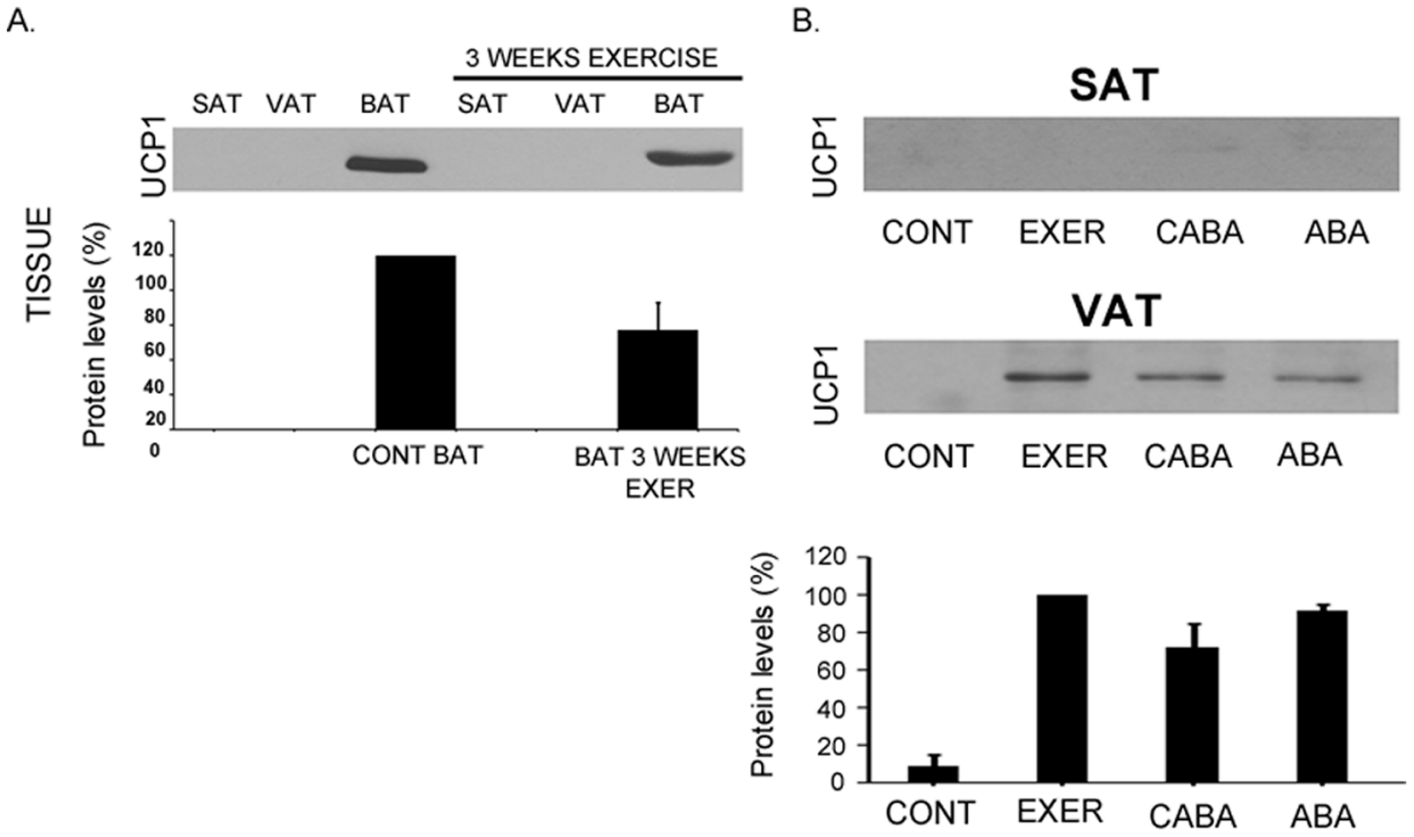
UCP1 detection in adipose tissue. Representative western blot images of UCP1 for SAT, VAT and VAT under ad libitum condition and after 3 weeks of exercise are shown (A); UCP1 expression in SAT and VAT among anorexic and control counterparts is shown (B). Histograms of band quantification from at least 3 independent experiments are shown in those cases in which bands exist. Significant protein results are shown as percentages compared with controls in A and with regard to EXER in B (*p<0.05; **p<0.01).

Considering the described potent effect of FNDC5/irisin stimulating white adipose browning and thermogenesis [1], we tested Ucp1 expression on the subcutaneous and visceral adipose tissues analyzed above (Figure 4). Under normal ad libitum conditions, Ucp1 was expressed exclusively in BAT as expected (Figure 4A). However, this expression shows a decreasing trend after 3 weeks of endurance exercise (Figure 4A).

In the case of pathological animal models, detection of Ucp1 expression in SAT and VAT adipose tissues from either obese or lean animals was not possible. The same lack of expression in SAT was also observed among ABA rats and their control counterparts (Figure 4B); however, a strikingly high expression was detected in VAT among exercised (EXER), fasting (CABA) or both (ABA) animals (Figure 4B).

### PGC1α protein expression

**Figure 5:**
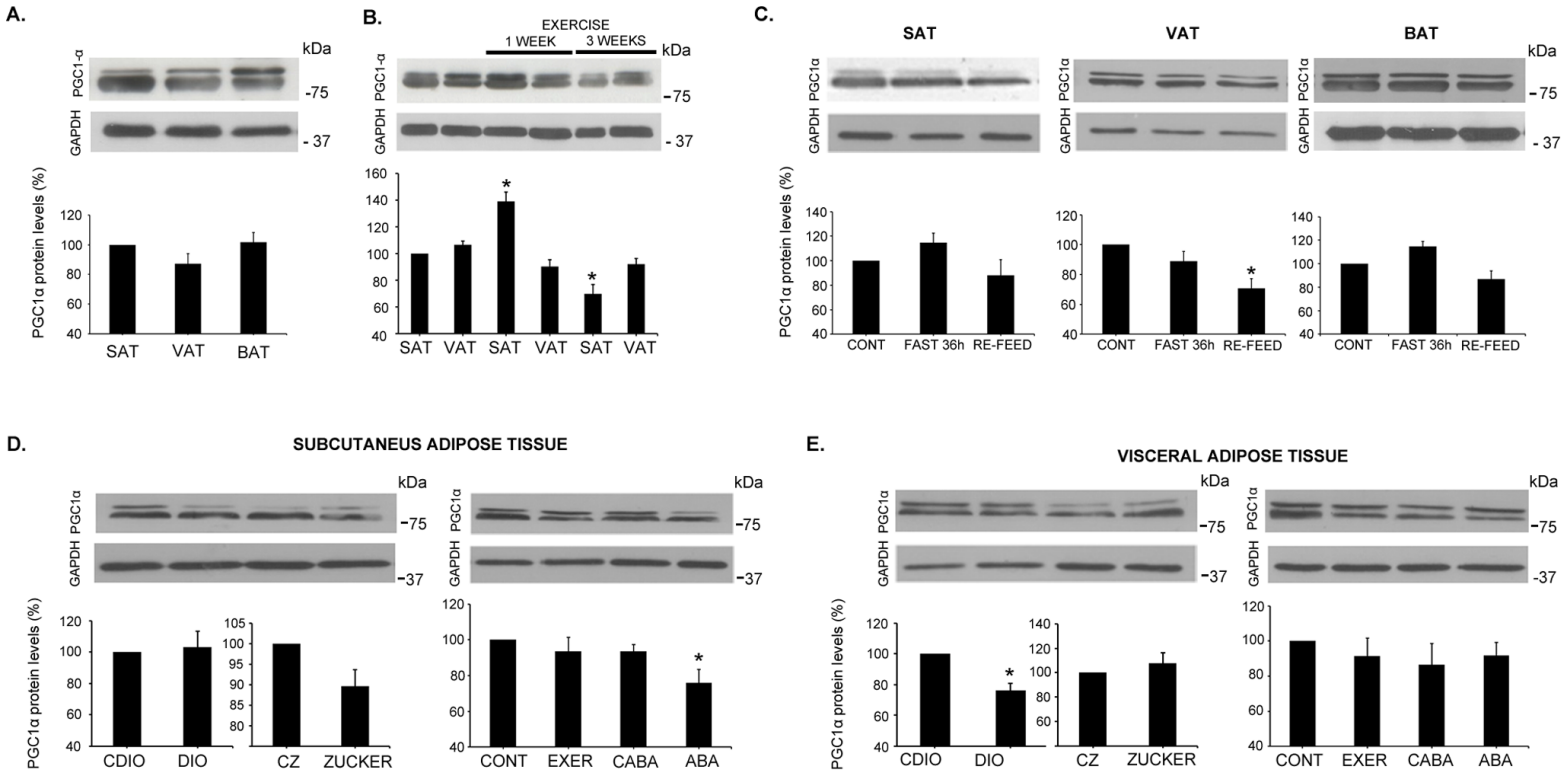
Adipose tissue expression of PGC1α. PGC1α expression was assessed in whole adipose tissue using western blot in the same secretion study samples shown in Figure 2. Representative images of western blots and histograms of band quantification towards GAPDH from at least 3 independent experiments are shown. Significant protein results are represented as percentages with regard to each control (*p<0.05). SAT: subcutaneous adipose tissue; VAT: visceral adipose tissue; BAT: brown adipose tissue; DIO: diet induced obesity; CDIO: control DIO; CZ (fa/-): control Zucker; Zucker: fa/fa; CONT: control; EXER: exercise; CABA: control ABA; ABA: activity based anorexia.

Given the implication of PGC1α in energy metabolism, and its role in activating FNDC5 in exercising muscle, we analyzed its expression at protein level in all the experimental situations established in our study (Figure 5). Generally, we did not find differences in PGC1α protein expression in WAT or BAT at different physiological or pathological situations (Figure 5A–E). However, we observed a significant increase of PGC1α in SAT after one week of exercise training; this elevation on PGC1α was reverted after longer periods of exercise (Figure 5B). In addition, SAT from anorexic animals and VAT from DIO showed a significant diminution of PGC1α (Figure 5D and 5E).

### FNDC5/Irisin is secreted by rat mature adipocytes and human adipose tissue

**Figure 6:**
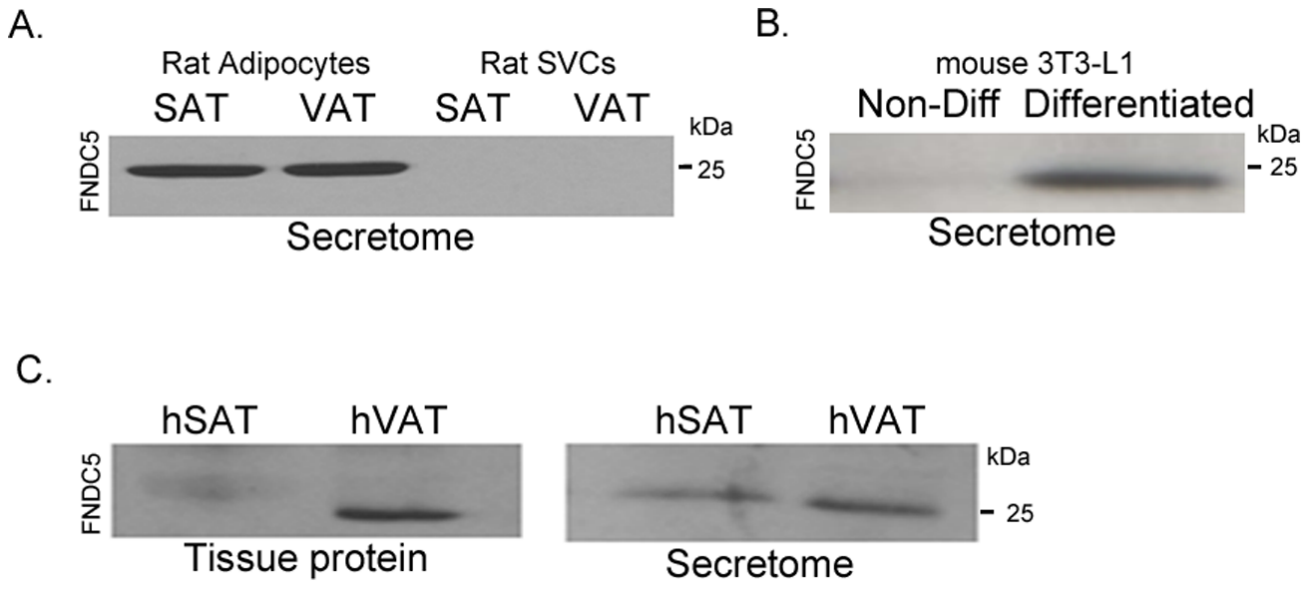
Mature adipocytes in rats and human adipose tissue secrete FNDC5. The occurrence of FNDC5 in rat and human adipocytes as well as SVCs secretomes (A); in differentiated and non-differentiated 3T3-L1 cell secretomes (B); and in human subcutaneous and visceral adipose tissues and secretomes (C).

In an attempt to better characterize the nature of cells that secrete irisin within adipose tissue, we assessed irisin secretion in fractionated SAT and VAT rat primary cell cultures. The immunoblots of the resulting secretomes confirmed that mature adipocytes are responsible for adipose tissue irisin secretion in both cases (Figure 6A). The differentiated 3T3-L1 cells also confirmed this result (Figure 6B). Finally, we found that human SAT and VAT also expressed and secreted FNDC5/irisin (Figure 6C).

## Discussion

The recent discovery of the PGC1α-dependent myokine FNDC5/irisin, which is able to stimulate brown-fat-like development and thermogenesis in white fat both in vitro and in vivo, has created a new field of research and many expectations in recent months [1], [22]–[27]. In this report, we are adding valuable novel information concerning the role of FNDC5/irisin by investigating the nature of its secretion, differences in muscle-tissue-type secretion, and its potential function as an adipokine expressed and secreted by WAT. Moreover, our results suggest that adipose-tissue-secreted FNDC5/irisin is influenced by circulating levels of this protein.

Although Boström and colleagues described the discovery of FNDC5/irisin and its potential role, certain aspects regarding this protein were not fully clarified in their manuscript or elsewhere [1]. They consider that FNDC5 is synthesized as a Type I membrane protein that is proteolytically cleavaged releasing the amino terminal portion of the 12 kDa peptide, into the extracellular space. To demonstrate the secretion of this myokine, however, they used an Abcam antibody directed against the endogenous portion of the protein (Abcam 149–178C-terminal). Using this antibody, they detected substantial amounts of FNDC5 at a molecular weight of at least 32 kDa due to glycosylation in the culture media of HEK 293 cells transfected with a vector that expressed the C-terminal flag-tagged FNDC5. Using the same antibody, however, they also detected a 22 kDa band in the plasma of mice that had been forced to express FNDC5 by the liver through the adenoviral intravenous delivery of a full-length FNDC5 vector. Thus, these evidences suggest that the full length FNDC5/irisin is also secreted, as recently annotated in the Uniprot protein Knowledgebase (UniProtKB Ref. Q8NAU1 Fibronectin Type III domain-containing Protein 5). Given this context, we believe that the present paper contributes to clarify this issue since we used the same antibody as Boström and colleagues against the endogenous form of the protein (anti-FNDC5 Abcam 149–178C-terminal), and an additional antibody directed against the theoretically soluble secreted form of 12 kDa (anti-Irisin Phoenix Pharmaceuticals amino acids 42–112) to characterize the FNDC5/irisin myokine regulation in rats under different nutritional and pathological conditions. Interestingly and in accordance with Boström's results, we detected a predominant band of approximately 25 kDa in both cases as well as extra bands of glycosylation, especially when using the anti-irisin antibody. This antibody also showed a faint band at approximately 12 kDa that could not be quantified. Thus, we analyzed the 25 kDa band in all our experiments. In the case of serum western blots, we detected extra bands of different intensities that might be the result of protein de-glycosylation at circulating level. To our knowledge, the direct secretion of the 12 kDa form of irisin from muscles or other tissues has not been described so far.

Regarding the experimental design chosen to analyze the role of FNDC5/irisin in the present work, it could be argued that contaminant or non-expected proteins might be present in the secretomes. The occurrence of unexpected proteins is related to unavoidable cell breakage and artifacts or to the ability of proteins to translocate between intra- and extracellular compartments in unconventional ways [28]–[30]. We have previously shown that these contaminants are constantly present in adipose tissue secretomes without finding any variation within their physiological or pathological state [14]. Thus we believe that the FNDC5/irisin protein detected in our western blots corresponds to muscle or adipose tissue active secretion because its levels are variable and comparable with previously published studies [1].

In the current study, we were unable to detect a significant increase of PGC1α expression in the exercised muscles of rats (after 3 weeks of voluntary wheel running followed by 12 hours rest before euthanasia), which parallels previous reports [8], [31]. Conversely, we observed a positive correlation between exercise and PGC1α expression levels in adipose tissue as previously described [32], which correlated with FNDC5/irisin secretion pattern. Nevertheless, this correlation was not found in those experimental situations different from exercise in our study. Thus, these results suggest an additional regulation of FNDC5 independent of PGC1α in adipose tissue.

Knowing the importance of the role of adipose tissue on energy homeostasis and its participation in obesity [33], [34], the discovery of FNDC5/irisin as a new adipokine described in our manuscript suggests an autocrine and most likely an endocrine function for this peptide in adipose tissue. Thus, given that approximately 72% of circulating FNDC5/irisin comes from muscle tissue [1], adipose-tissue-secreted FNDC5/irisin might participate in the remaining 28%. According to our previous analysis on adipose tissue secretion [14], FNDC5/irisin shows a distinct pattern of secretion that depends on the anatomical location of fat. Thus, the fact that SAT secretes much more FNDC5/irisin than other fat deposits is expected after accounting for the “beneficial” role of this type of tissue compared with visceral fat, which has been shown to participate in the metabolic complications associated with weight gain and obesity [35], [36]. In relation to exercise, it is of interest the early pattern of secretion observed in adipose tissue compared to muscle [1]. Our results indicate that WAT increases FNDC5/irisin secretion with short-term exercise (1 week), whereas 3 weeks of exercise significantly decrease FNDC5/irisin secretion. Many questions must be answered regarding the role of adipose-FNDC5/irisin; however, if FNDC5/irisin it is able to drive WAT into BAT as indicated by Boström [1], it seems that it is not in the interest of WAT to stimulate thermogenesis and energy consumption under prolonged exercise. Consequently, we observed that 36 hours of fasting reduces FNDC5/irisin secretion in WAT. Accordingly, the CABA animals that were subjected to severe fasting showed a decrease of FNDC5/irisin secretion, especially in VAT. Although the effect of fasting on direct FNDC5/irisin secretion by muscle tissue remains unknown, the circulating FNDC5/irisin levels observed in our experiment suggest that fasting induces muscle secretion of this protein. Thus, we analyzed possible WAT browning by assaying the BAT-specific uncoupling protein 1 (Ucp1) responsible for nonshivering thermogenesis to maintain body temperature [37]. Unexpectedly, we detected Ucp1 expression in VAT among anorexic animals and their control counterparts with the same exercise training (EXER) or food restriction (CABA). The Ucp1 expression in these cases parallels the FNDC5/irisin circulating levels. Moreover, we observed that Ucp1 expression in VAT disappears after 3 weeks of exercise. In this regard, Boström and collaborators described a great amount of browning in subcutaneous fat and, to a minor extent, also in visceral fat after 3 weeks of wheel running [1]. We believe that our findings concerning VAT are not comparable with those described by Boström because these authors used white adipose tissue from the epididymis as visceral tissue. In our experiments, we chose the WAT located inside the peritoneal cavity around the internal organs, which we believe better resembles the human visceral fat close to the liver. Moreover, we previously demonstrated that rat gonadal fat from the epididymis shows a different pattern of protein secretion compared with the visceral fat in the peritoneal cavity [14]. Nevertheless, we were unable to detect Ucp1 expression after three weeks of exercise in our experimental model.

The liver-forced expression of FNDC5 and the consequent increase of FNDC5/irisin circulating levels significantly improved glucose tolerance and reduced fasting insulin in obese and insulin-resistant mice according to Boström and colleagues [1]. In our study, however, we detected an over-secretion of FNDC5/irisin in the WAT of obese animals. Although Boström et al. did not report the grade of FNDC5/irisin secretion by muscle in obese compared to lean animals; we detected a significant elevation of circulating FNDC5/irisin in DIO animals. Our result suggests that animals with exposure to high levels of FNDC5/irisin might develop a resistance to this protein. If this hypothesis is true, the unquestionable role of muscle stimulating several of the beneficial effects of exercise would need to be explained by alternative mechanisms.

Globally, FNDC5/irisin showed a secretion pattern similar to other well-known adipokines (e.g., leptin); thus, our results also suggest a feedback pattern in which adipose tissue might be sensitive to FNDC5/irisin circulating levels. Because adipose tissue is determined to preserve its energy saving function to maintain energy balance, it stands to reason to find that elevated levels of circulating FNDC5/irisin correspond to decreased levels of irisin secretion by adipose tissue of the studied animals. Under this situation, irisin resistance might explain the high secretion pattern of WAT observed in the DIO animals; however, further research should be performed to confirm this hypothesis. In view of the fact that Zucker obese rats with no functional leptin receptor seem not to be affected by circulating FNDC5/irisin levels, other mechanisms related to leptin might be also implicated.

Recently, Huh and collaborators published a report about the role of circulating irisin and FNCD5 in humans [38]. Contrary to our results, they found very low mRNA expression of FNDC5 in human fat compared with muscle using a TissueScan qPCR Array. Generally, mRNA expression is assumed to be predictive of protein expression level; importantly, however, the correlation between mRNA and protein expression has been described as moderately or weakly positive (correlation coefficients ranging from 0.2 to 0.6), especially with regard to secreted proteins [39], [40]. Our results illustrate this fact well; thus, the correlation between FNDC5/irisin mRNA levels and secretion levels was not always evident. Several independent considerations should be taken into account with regard to the data published by Huh and collaborators concerning the nature and origin of their samples which makes comparisons with our data difficult.

On the other hand, a recent study performed by Stengel and collaborators strongly supports our results [41]. These authors observed higher circulating irisin levels in obese patients compared with normal weight and anorexic patients, which resulted in a correlation between irisin and body weight and fat mass among other variables. These data suggest that circulating irisin is affected under conditions of altered BMI, with the highest levels in obese patients. Taking these premises, and based in our direct explants secretion studies, we postulate that the muscle/adipose irisin secretion ratio might vary by the physiological situation. Thus, with exercise training, muscle tissue would strongly affect the FNDC5 circulating levels, whereas in atypical BMI cases such as obesity, adipose tissue would actively elevate FNDC5/irisin.

Our data sets the basis for additional research since many questions remain to be answered concerning FNDC5/irisin such as the nature of the FNDC5/irisin receptor (which is yet to be discovered) and the precise role of the soluble long and short forms of FNDC5/irisin. In conclusion, this study reveals that rat skeletal muscle sheds the long form of FNDC5 whose secretion depends on the muscle fiber type. More interestingly, this work is the first to identify FNDC5/irisin as a potential adipokine secreted by WAT and especially by subcutaneous adipose tissue. Given that exercise, nutritional status and pathological conditions such as obesity influence WAT-secreted FNDC5/irisin, our findings suggest a regulatory mechanism influenced by circulating FNDC5/irisin in a feedback process.

## Supporting Information

Table S1 **After 5 days of acclimatization, the weight-matched animals were assigned to one the following experimental groups (n = 5 per group).** a) control ad libitum (CONT), b) 36 hours fasting (FAST 36 h), c) re-feed after 36 hours fasting followed by 15 minutes of feeding (RE-FEED), d) exercise ad libitum with free access to the activity wheel for 1 or 3 weeks (EXER), e) control activity-based anorexia (CABA), and f) ABA as previously described (Routtenberg and Kuznesof 1967; Pardo et al. 2010). Diet-induced obesity (DIO) animals (200 g) were fed with a 60% high fat diet D12492 (Research Diets, NJ) over 9 weeks, and age-matched lean rats were used as a control. Male obese Zucker rats (fa/fa, n = 10) and their control counterparts (CZ), lean Zucker rats (fa/-, n = 10), were purchased from Charles River Laboratories (Barcelona, Spain) at 10 weeks of age and fed chow ad libitum for 12 weeks.
(DOCX)
