# Supplementary material for: FNDC5/Irisin Is Not Only a Myokine but Also an Adipokine
Source: PLoS One. 2013 Apr 11;8(4):e60563. doi: 10.1371/journal.pone.0060563 (PMC3623960; doi:10.1371/journal.pone.0060563)
Supplement: Table S1 — After 5 days of acclimatization, the weight-matched animals were assigned to one the following experimental groups (n = 5 per group). a) control ad libitum (CONT), b) 36 hours fasting (FAST 36 h), c) re-feed after 36 hours fasting followed by 15 minutes of feeding (RE-FEED), d) exercise ad libitum with free access to the activity wheel for 1 or 3 weeks (EXER), e) control activity-based anorexia (CABA), and f) ABA as previously described (Routtenberg and Kuznesof 1967; Pardo et al. 2010). Diet-induced obesity (DIO) animals (200 g) were fed with a 60% high fat diet D12492 (Research Diets, NJ) over 9 weeks, and age-matched lean rats were used as a control. Male obese Zucker rats (fa/fa, n = 10) and their control counterparts (CZ), lean Zucker rats (fa/-, n = 10), were purchased from Charles River Laboratories (Barcelona, Spain) at 10 weeks of age and fed chow ad libitum for 12 weeks. (DOCX) [file pone.0060563.s001.docx]

**Table S1: Animal Models**

| **Animal Model^1^** | **Feeding regime** | **Exercise Training** |
| --- | --- | --- |
| Control *ad libitum* (n=5) | *Ad libitum* chow diet | None |
| Fasting (n=5) | 36 hours fasting | None |
| Re-feed (n=5) | 36 hours fasting followed by 15 minutes of re-feeding | None |
| Exercise (n=5) | *Ad libitum* chow diet | 1 or 3 weeks of wheel running |
| CABA (n=5) | Same feeding regime as ABA | None |
| ABA (n=5) | 1 hour chow diet feeding each day for 1 week and free access to water | 1 week of wheel running |
| CDIO (n=10) | *Ad libitum* chow diet for 9 weeks | None |
| DIO (n=10) | 60% high fat diet D12492 (Research Diets, NJ) for 9 weeks | None |
| CZ (fa/-, n=10) | *Ad libitum* chow diet for 12 weeks | None |
| Zucker (fa/fa, n=10) | *Ad libitum* chow diet for 12 weeks | None |
